# Caregiver Burden Associated With Respiratory Syncytial Virus (RSV) Infection in Young Children in Japan

**DOI:** 10.7759/cureus.91502

**Published:** 2025-09-02

**Authors:** Shinichi Noto, Naoki Shimizu, Ayumi Morishita

**Affiliations:** 1 Rehabilitation, Niigata University of Health and Welfare, Niigata, JPN; 2 Vaccine Public Affairs, Sanofi K.K., Tokyo, JPN; 3 Health Economics and Outcomes Research, Real World Evidence Solutions, IQVIA Solutions G.K., Tokyo, JPN

**Keywords:** caregiver burden, immunization, japan, prevention, respiratory syncytial virus

## Abstract

Introduction and aim: Respiratory syncytial virus (RSV) not only affects the infected child but can also affect the quality of life experienced by caregivers and families. Evidence is limited on the impact of caring for RSV-infected children. This study aimed to explore caregivers' experiences, considering the unique cultural aspects of Japan.

Methods: A sequential exploratory mixed-methods approach was employed. Mothers and fathers with children who contracted RSV before the age of one were recruited via a patient panel. Caregivers were defined as those who provided direct care to the child during their RSV infection. RSV diagnosis was based on caregiver self-report; physician confirmation was not required. Semi-structured interviews with caregivers were first conducted to elicit relevant concepts and questions. The final questionnaire was administered to caregivers in September 2023. The questionnaire assessed emotional, physical, and financial impacts, as well as the impact on and motivation to work while caring for their RSV-infected child.

Results: A total of 309 caregivers were included in the study. Caregivers reported a decrease in sleep duration and health quality after their child contracted RSV. Over 40% (53/120) of fathers felt the most significant time loss was due to work, while over 30% (61/189) of mothers felt it was due to house chores. Both parents felt emotionally burdened by seeing their child suffer and were anxious about their child's symptoms and conditions. More than 60% (148/235) of working parents had to adjust their work style, with some feeling mentally burdened from taking leave or being unable to take leave from work. Additionally, more than 60% (145/235) experienced changes in their perception towards work. Notably, 48.3% (58/120) of fathers perceived themselves as the primary caregiver, an unexpected result given the traditional gender roles of caregiving in Japan.

Conclusion: This was a mixed-methods study in Japan assessing the caregiver burden of RSV-infected children. The findings provide an important perspective illustrating the impact of RSV infection and supporting the importance of adequate disease prevention.

## Introduction

Respiratory syncytial virus (RSV) is the most common cause of lower respiratory tract infections. While primarily causing mild, cold-like symptoms, its impact can be serious, particularly among infants and young children, in whom it manifests primarily as pneumonia and bronchiolitis [1]. RSV infection, while most prevalent in children younger than one, contributes greatly to morbidity and mortality in children under five years of age and has implications for long-term respiratory health [2,3]. Severe cases require hospital admission, and infected children are at an increased risk of developing chronic conditions, including recurrent wheezing and asthma later in their lives [3-5].

In Japan, the incidence rate of medically attended RSV and RSV hospitalization among Japanese children below two years of age is 85.2 per 1,000 person-years and 23.2 per 1,000 person-years, respectively [6]. The mortality rate of RSV infection is less than 1% but reaches 19% in patients with severe infections [7]. While general RSV-related hospitalizations are already financially taxing, intensive care unit hospitalizations are associated with even greater costs [8,9]. Therefore, RSV has a considerable impact on the healthcare system and population in Japan.

Furthermore, RSV infection has a serious impact not only on children but also on the infected infants’ caregivers. Studies conducted in Europe and the United States of America (USA) show that the caregivers of children hospitalized with RSV may need to miss work and suffer productivity loss when at work [10-12]. Meanwhile, a significant emotional impact was observed among caregivers of children infected with RSV. Having a child hospitalized due to RSV may cause higher levels of stress, more anxiety, and poorer family functioning [13]. Disruption of sleep and family routines, separation from siblings, and strain in finances and family relationships were also reported [11].

Despite being globally acknowledged, the impact of RSV on caregivers in Japan is not well understood. A clearer understanding of caregiver experiences, including their emotional, physical, and financial dimensions, is essential for shaping responsive healthcare policies and developing support strategies that address the practical and psychological challenges caregivers face in Japan. To address this gap, we conducted an exploratory sequential mixed-methods study, combining semi-structured interviews with a follow-up questionnaire survey, to comprehensively describe the experiences of caregivers of children diagnosed with RSV before the age of one year.

## Materials and methods

Study design

This non-interventional, cross-sectional study was conducted in Japan using a mixed-methods approach to explore the experience of caregivers of children under one year of age infected with RSV. Caregivers were defined as mothers or fathers of RSV-infected children who provided direct care at the time of infection. RSV diagnosis was based on caregiver self-report; physician confirmation was not required. To support the integration of qualitative and quantitative components in this mixed-methods study, convenience sampling was employed to ensure continuity between the exploratory (qualitative) and confirmatory (quantitative) phases.

The qualitative phase involved semi-structured interviews with 11 caregivers, conducted between April 12 and April 28, 2023. Interviews were conducted in the Japanese language and moderated by trained interviewers using a standardized guide. They were audio- and/or video-recorded with participant consent and transcribed verbatim. Transcripts were reviewed for accuracy and analyzed thematically to identify key burden domains, which informed the development of the quantitative survey. A single coder conducted a thematic analysis, while another researcher created case summaries. The findings were then integrated into a report that combined cross-case themes and individual narratives.

The quantitative phase consisted of a web-based, self-administered questionnaire in Japanese, conducted from September 4 to 5, 2023. The survey included items derived from the qualitative findings and assessed the impact of RSV on caregivers across physical, emotional, and financial domains.

To confirm RSV infection in their children, participants were asked the following screening questions: (1) “Has your child(ren) ever contracted RSV?” and (2) if the answer was yes and multiple children had been infected, “What was the child’s age when he or she contracted RSV?” Participants were instructed to respond based on their youngest child who had experienced RSV, regardless of whether the care was inpatient or outpatient, in a clinic or hospital. 

Recruitment, enrollment, and variables

Participants were recruited via a patient panel managed by Macromill Carenet Inc., a market research company specializing in the healthcare sector in Japan, which confirmed the largest sample sizes for both qualitative and quantitative phases among the patient panel providers we had considered [14]. Consistent with approaches used in similar studies, we employed a convenience sampling strategy to ensure representation of caregivers [15,16]. In this study, the inclusion criteria consisted of those who were Japanese adults aged 18 years and older who had experienced the burden of caring for a child diagnosed with RSV before their first birthday. The survey was administered online through Macromill’s proprietary platform. Caregivers engaged in or affiliated with the pharmaceutical industry, as well as those who had difficulty understanding Japanese, were excluded to ensure the study's integrity.

All participants provided electronic consent before entering the surveys through their smartphones, tablet devices, or computers. In the qualitative phase, a total of 11 participants were enrolled through an online panel. The sample size was determined with reference to the Turner-Bowker qualitative interview saturation point, which supports the identification of key burden domains with a limited number of interviews [17,18]. To minimize recall bias, priority was given to participants with shorter recall periods. These interviews were conducted to explore caregiver experiences, and the findings directly informed the development of the quantitative questionnaire used in the subsequent phase.

In the quantitative phase, caregivers were recruited through an established online panel to complete a self-administered, web-based survey. The sample size of 300 was determined based on feasibility and the objective of generating a comprehensive descriptive overview of caregiver burden. The survey instrument was developed using insights from the qualitative phase to ensure conceptual continuity and relevance (appendix). The data collected included caregiver sociodemographic characteristics (e.g., age, gender, caregiver type, employment status), the child’s health status (e.g., symptom duration, care setting, comorbidities, use of RSV prophylaxis), and multiple dimensions of caregiver burden. These dimensions encompassed emotional, physical, and financial impacts, as well as changes in daily life, time lost to caregiving, support received, and effects on work and motivation. Financial burden was assessed in terms of household expenses, recognizing that direct medical costs are largely covered by Japan’s national and private health insurance systems. As the questionnaire was grounded in qualitative data, no formal validation study or pilot testing was conducted prior to its use.

Similar to the qualitative phase, caregivers with a recall period of less than five years were prioritized to enhance the accuracy of their responses. As a result, 382 participants met the eligibility criteria and responded to the survey. After excluding incomplete and invalid responses, 309 participants who fully completed the questionnaire were included in the final analysis.

Statistical methods

Participant characteristics and responses regarding emotional, physical, and financial burdens were summarized using means, standard deviations (SD), medians, and interquartile ranges (IQR) for continuous variables, and total numbers and percentages per category for categorical variables. All statistical analyses were performed using Microsoft Excel (Redmond, WA: Microsoft Corp.).

Ethics approval and consent to participate

The study protocol and related study documents were reviewed by the Shiba Palace Clinic Ethics Committee for ethical and scientific appropriateness of the study, and ethics approval was obtained on February 22, 2023 (approval #151505_rn-34402) [19]. Electronic consent was obtained from all study participants before they completed the online survey. The entire study process complies with the Ethical Guidelines for Medical and Health Research Involving Human Subjects.

## Results

Participant demographics and characteristics

A total of 309 caregivers (189 mothers and 120 fathers), aged 25-59 years, with children who contracted RSV at a mean (SD) age of 6.7 (3.7) months, participated in this study (Table 1). Approximately three-quarters (74.8%, n=231) of the 309 participants considered themselves primary caregivers of their children, among whom 58 (25.1%) were fathers and 173 (74.9%) were mothers. Of the 120 male respondents who participated in the survey, 48.3% (n=58) identified themselves as the primary caregiver, while 91.5% (173/189) of the female respondents considered themselves the primary caregiver. Descriptively, more mothers tended to be on parental leave compared to fathers (25.4% and 3.3%, respectively). The median current age of the children was 2.7 years (IQR: 1.3-4.3).

**Table 1:** Demographic information of 309 participating caregivers and their children. ^1^This was a multiple-choice question. ^2^Employment status (on parental leave; full-time/short-time employee; contract/part-time worker; homemaker/unemployed) further stratified by mothers (n=48, 25.4%; n=36, 19.0%; n=32, 16.9%; n=73, 38.6%) and fathers (n=4, 3.3%; n=112, 93.3%; n=3, 2.5%; n=1, 0.8%). ^3^Participants were asked if their RSV-infected child had any of the following conditions at disease onset: pulmonary hypoplasia; airway stenosis; congenital esophageal atresia; congenital metabolic disorder; neuromuscular disease; asthma; bronchitis; heart disease; lung disease; immunodeficiencies; low birthweight; or trisomy 21 (Down syndrome). ^4^Primary caregivers were further stratified by mothers (n=173, 74.9%) and fathers (n=58, 25.1%). One household may include multiple primary caregivers. ^5^“Not applicable” was a possible response option to the question regarding underlying conditions at disease onset. RSV: respiratory syncytial virus

Characteristics		Participants
Demographic information of caregivers		
Caregiver type, n (%)	Primary^4^	231 (74.8)
	Non-primary	78 (25.2)
Gender of caregiver, n (%)	Father	120 (38.8)
	Mother	189 (61.2)
Age of caregiver in years, n (%)	25-29	27 (8.7)
	30­­-34	80 (25.9)
	35-39	113 (36.6)
	40-44	57 (18.5)
	45-49	24 (7.8)
	50-54	7 (2.3)
	55-59	1 (<0.1)
	60 and above	0 (0)
Current age of child (in years)	Mean (SD)	2.8 (1.8)
	Median (IQR)	2.7 (1.3-4.3)
Demographics of caregivers at the time of RSV infection		
Support from people other than spouse/partner at disease onset^1^, n (%)	Parents	145 (46.9)
	Siblings	20 (6.5)
	Friends	6 (1.9)
	Neighbors, community support	9 (2.9)
	Other relatives, etc.	10 (3.2)
	None	148 (47.9)
Employment status at disease onset^2^, n (%)	On parental leave	52 (16.8)
	Full-time/short-time employee	148 (47.9)
	Contract/part-time worker	35 (11.3)
	Homemaker/unemployed	74 (24.0)
Condition of child at the time of RSV infection		
Age of child at disease onset (in months)	Mean (SD)	6.7 (3.7)
	Median (IQR)	7.0 (4.0-10.0)
Duration of symptoms of RSV infection, n (%)	Within 3 days	77 (24.9)
	4 days to less than a week	132 (42.7)
	1-4 weeks	65 (21.0)
	1-6 months	9 (2.9)
	Over 6 months	2 (0.7)
	No symptom	24 (7.8)
Status of hospitalization of child, n (%)	Inpatient (accompanied by caregiver)	80 (25.9)
	Inpatient (unaccompanied by caregiver)	17 (5.5)
	Outpatient - with follow-up visits	106 (34.3)
	Outpatient - single visit, no follow-up	106 (34.3)
Underlying conditions at disease onset^3^, n (%)	Yes	57 (18.5)
	Not applicable^5^	252 (81.6)
Use of preventive option at disease onset, n (%)	Yes	104 (33.7)
	No	205 (66.3)

In approximately 70% (n=209) of cases, symptoms resolved within less than a week, with more than 60% (n=132) resolving within four to seven days among them. However, symptoms lasted over a month in 11 cases (3.6%) and one to four weeks in 65 cases (21.0%), showcasing great variability in the duration of disease symptoms. Over two-thirds (n=212, 68.6%) of all caregivers reported having received outpatient care only, with half of them taking their child back to the doctor for follow-up visits. About a quarter (n=80, 25.9%) of all caregivers experienced an overnight hospital stay with their children. While 52.1% (n=161) of the participants received some support from their social network, most of which came from their parents (n=145, 46.9%), 47.9% (n=148) reported having no support at all. Approximately one-third (n=104, 33.7%) of caregivers reported having used RSV prophylaxis at the time of disease onset. Fewer than one-fifth (n=57, 18.4%) reported that their child had an underlying condition required to be eligible for the use of RSV prophylaxis.

Burden related to time and work

More than a quarter of all caregivers felt they had lost time for household chores (n=82, 26.5%) and work (n=80, 25.9%) due to caring for their child with RSV (Table 2). Notably, a higher proportion of mothers than fathers indicated a loss of “time for household chores” (32.3% vs. 17.5%), whereas almost half of the fathers reported a loss of “time for work” (44.2%). Additionally, a considerable percentage of caregivers identified a loss of “time for hobbies and personal relaxation” (n=57, 18.4%) and “sleep time” (n=50, 16.2%).

**Table 2:** Impact on caregivers’ time and work due to child’s RSV infection. ^1^The multiple-choice questions were displayed to participants who answered that they had been working or were on parental leave (i.e., who chose answer options other than homemaker or unemployed) at the time their child contracted RSV. ^2^The question was displayed to participants who answered that they had been working (i.e., who chose answer options other than homemaker, unemployed, or parental leave) at the time their child contracted RSV. RSV: respiratory syncytial virus

Types of time lost most from caring for child with RSV, n (%)	Total (n=309)	Mother (n=189)	Father (n=120)
Time for work	80 (25.9)	27 (14.3)	53 (44.2)
Time for household chores	82 (26.5)	61 (32.3)	21 (17.5)
Time for hobbies and personal relaxation	57 (18.4)	37 (19.6)	20 (16.7)
Time for studying qualifications or self-improvement	6 (1.9)	3 (1.6)	3 (2.5)
Sleep time	50 (16.2)	35 (18.5)	15 (12.5)
Time for taking care of siblings	13 (4.2)	13 (6.9)	0 (0)
Not applicable	21 (6.8)	13 (6.9)	8 (6.7)
Adjustments balancing work and caring for an infected child^1^, n (%)	Total (n=235)	Mother (n=116)	Father (n=119)
Took paid leave and nursing leave	112 (47.7)	44 (37.9)	68 (57.1)
Took a leave of absence	16 (6.8)	12 (10.3)	4 (3.4)
Resigned from the job	8 (3.4)	2 (1.7)	6 (5.0)
Changed workplace	6 (2.6)	0 (0)	6 (5.0)
Changed work arrangements (such as working part-time or from home)	21 (8.9)	9 (7.8)	12 (10.1)
Consulted with the workplace and colleagues to make adjustments	21 (8.9)	9 (7.8)	12 (10.1)
No change	87 (37.0)	52 (44.8)	35 (29.4)
Changes in perception or attitude towards work^1^, n (%)	Total (n=235)	Mother (n=116)	Father (n=119)
Losing motivation for work	37 (15.7)	11 (9.5)	26 (21.8)
Finding it difficult to continue working	51 (21.7)	27 (23.3)	24 (20.2)
Becoming hesitant to take on responsible positions or major roles	38 (16.2)	11 (9.5)	27 (22.7)
Becoming anxious about balancing parenting and work	69 (29.4)	43 (37.1)	26 (21.8)
Not applicable	90 (38.3)	44 (37.9)	46 (38.7)
Mentally burdened from taking leave or being unable to take leave^2^, n (%)	Total (n=183)	Mother (n=68)	Father (n=115)
Totally agree	45 (24.6)	20 (29.4)	25 (21.7)
Somewhat agree	83 (45.4)	27 (39.7)	56 (48.7)
Somewhat disagree	36 (19.7)	15 (22.1)	21 (18.3)
Totally disagree	19 (10.4)	6 (8.8)	13 (11.3)

A total of 235 caregivers were employed (i.e., working or on parental leave) at the time of infection. About two-thirds (n=148, 63.0%) of these caregivers reported having made adjustments to balance work and care for the infected child. Among these caregivers, most of the adjustments included taking paid leave and nursing leave (n=112, 47.7%), changing work arrangements (n=21, 8.9%), or consulting with colleagues (n=21, 8.9%). However, some caregivers had to go as far as changing workplaces (n=6, 2.6%) or resigning from their jobs (n=8, 3.4%). These adjustments were reported by both mothers and fathers, with a high proportion of fathers adjusting their work to care for their sick child. Perception or attitude towards work was also affected, with 37.1% (n=43) of mothers becoming anxious about balancing parenting and work and 23.3% (n=27) finding it difficult to continue working; whereas 22.7% (n=27) of fathers felt hesitant to take on more responsibilities or major roles at work, lost motivation towards work (n=26, 21.8%), became anxious about balancing parenting and working (n=26, 21.8%), or found it difficult to continue working (n=24, 20.2%).

Among the 183 caregivers (115 fathers and 68 mothers) who worked and were not on parental leave at the time their child contracted RSV, 70.4% (n=81) of fathers and 69.1% (n=47) of mothers reported experiencing mental burden due to having to adjust their work schedules or being unable to take time off to care for their children.

Emotional burden

Table 3 shows the emotions that were felt strongly upon seeing their child suffer from an RSV infection among 290 caregivers who experienced emotional stress. Nearly three-quarters of caregivers (n=211, 72.3%) felt sympathetic seeing their child suffer, and more than half felt anxious about the duration of their child’s condition (n=164, 56.6%) and the prognosis of the disease (n=163, 56.2%). A greater proportion of mothers reported anxiety than fathers, whereas fathers were more likely to blame themselves for their child’s suffering than mothers (41.4% and 31.3%, respectively).

**Table 3:** Emotional burden of caregiving for a child suffering a RSV infection. ^1^The multiple-choice question was not displayed to a portion of participants (n=19) who answered “not applicable” in a previous question asking if they felt any cause of emotional stress when their child contracted RSV. RSV: respiratory syncytial virus

Feelings^1^, n (%)	Total (n=290)	Mother (n=179)	Father (n=111)
Feeling sympathy and pity for the child’s suffering	211 (72.8)	138 (77.1)	73 (65.8)
Blaming self for the young child’s illness	102 (35.2)	56 (31.3)	46 (41.4)
Feeling anxious about how long the breathing difficulties will continue	164 (56.6)	111 (62.0)	53 (47.7)
Feeling anxious about what will happen if the condition worsens	163 (56.2)	115 (64.2)	48 (43.2)
Feeling anxious about the possibility of other symptoms or complications arising	97 (33.4)	62 (34.6)	35 (31.5)
Feeling a sense of loneliness and feeling like there’s no one to rely on	30 (10.3)	22 (12.3)	8 (7.2)
Other feelings	3 (1.0)	3 (1.7)	0 (0)

Sleep duration and health quality

Perceived changes in sleep and health condition were prominent in all caregivers, with both mothers and fathers feeling their sleep time was reduced to nearly half and that their health status had worsened compared to the time before their child contracted RSV (sleep metric median {IQR}: 50 {40-70}; health metric median {IQR}: 60 {50-80}) (Table 4). Results also show that some caregivers had no sleep at all or that their health quality dropped in comparison to their sleep and health prior to the RSV infection.

**Table 4:** Changes in sleep and health from caring for child with RSV. ^1^Participants compared their sleep duration and health quality during their child's infection to before the RSV infection. Responses were recorded on a scale from 0 (no sleep/very poor health) to 100 (pre-infection sleep duration/health quality). RSV: respiratory syncytial virus

Sleep metrics^1^ (0-100)	Total (n=309)	Mother (n=189)	Father (n=120)
Mean (SD)	52.3 (24.99)	47.9 (24.4)	59.3 (24.1)
Median (IQR)	50.0 (40.0-70.0)	50.0 (30.0-60.0)	60.0 (50.0-80.0)
Health metrics^1^ (0-100)	Total (n=309)	Mother (n=189)	Father (n=120)
Mean (SD)	59.8 (24.3)	56.6 (24.2)	64.8 (23.6)
Median (IQR)	60.0 (50.0-80.0)	60.0 (50.0-80.0)	70.0 (50.0-80.0)

Financial burden

About half of all caregivers (n=151, 48.9%) recalled paying unexpected non-medical and medical fees (Table 5). Overall, the most frequently paid expenses were the cost of food for individuals other than the sick child or accompanying caregiver (n=47, 15.2%), caregiver’s meal expenses during the hospital stay (n=40, 12.9%), and private room fees at the hospital (n=38, 12.3%). Most non-medical costs, such as expenses for food, household chores, and childcare, were reported by caregivers whose child received inpatient care compared to those whose child received only outpatient care. A decrease in income was also reported in 41.1% (n=127) of overall caregivers but was most prominent in those whose child received inpatient care at over 56.0%.

**Table 5:** Unexpected expenses due to child's RSV infection. ^1^This was a multiple-choice question. RSV: respiratory syncytial virus

Unexpected expenses other than direct medical fees^1^, n (%)	Total (n=309)	Inpatient care (n=97)	Outpatient care (n=212)
Hospital private room fee	38 (12.3)	38 (39.2)	-
Caregiver's meal expenses during hospital stay	40 (12.9)	40 (41.2)	-
Purchase of medical equipment such as a nebulizer	36 (11.7)	20 (20.6)	16 (7.5)
Childcare expenses for the sick child	18 (5.8)	8 (8.2)	10 (4.7)
Childcare expenses for siblings	20 (6.5)	8 (8.2)	12 (5.7)
Expenses to outsource domestic services	17 (5.5)	10 (10.3)	7 (3.3)
Food expenses for individuals other than the accompanying caregivers and sick child	47 (15.2)	24 (24.7)	23 (10.8)
Other expenses	33 (10.7)	11 (11.3)	22 (10.4)
No unexpected expenses	158 (51.1)	13 (13.4)	145 (68.4)
Income decreases due to RSV infection of child, n (%)	Total (n=309)	Inpatient care (n=97)	Outpatient care (n=212)
Totally agree	44 (14.2)	23 (23.7)	21 (9.9)
Somewhat agree	83 (26.9)	32 (33.0)	51 (24.1)
Somewhat disagree	69 (22.3)	19 (19.6)	50 (23.6)
Totally disagree	113 (36.6)	23 (23.7)	90 (42.5)

Burden specific to caregivers of a child receiving inpatient care

Table 6 shows that for 97 caregivers whose child received inpatient care, the primary burden was associated with the hospital environment and overnight stays. These included inadequate sleeping conditions (n=50, 51.5%) and difficulty finding food (n=37, 38.1%) at the hospital, as well as heightened anxiety about the duration of hospitalization (n=41, 42.3%) and childcare for siblings (n=34, 35.1%), which were reported by a greater proportion of mothers than fathers. The most frequently reported burden among both mothers and fathers was witnessing their child in distress (60.0% among mothers and 42.9% among fathers).

**Table 6:** Caregiver burden specific to children receiving inpatient care. ^1^This multiple-choice question was displayed to participants who answered that their child was admitted to the hospital upon contracting RSV. RSV: respiratory syncytial virus

Caregiver burden during hospitalization^1^, n (%)	Total (n=97)	Mother (n=55)	Father (n=42)
Lack of meal options for self while accompanying child for hospital stay	37 (38.1)	24 (43.6)	13 (31.0)
Unable to sleep well from sharing a bed with child or using makeshift bed	50 (51.5)	32 (58.2)	18 (42.9)
Worrying about siblings while accompanying child for hospital stay	34 (35.1)	23 (41.8)	11 (26.2)
Unable to manage household chores	28 (28.9)	18 (32.7)	10 (23.8)
Seeing the child in distress	51 (52.6)	33 (60.0)	18 (42.9)
Difficulty in communication with doctors and nurses	13 (13.4)	6 (10.9)	7 (16.7)
Anxiety about the duration of hospitalization	41 (42.3)	30 (54.5)	11 (26.2)
Financial concerns including hospitalization costs	30 (30.9)	16 (29.1)	14 (33.3)
Anxiety about the workplace and job	13 (13.4)	5 (9.1)	8 (19.0)
Unable to meet the child who is hospitalized	10 (10.3)	4 (7.3)	6 (14.3)
Not applicable	5 (5.2)	3 (5.5)	2 (4.8)

## Discussion

Summary

This study adds to the scarce literature surrounding the caregiver burden of RSV-infected children in Japan, and it utilizes methods to improve the content validity of the administered questionnaire. The study found that caregivers of children infected with RSV experience a high burden emotionally, physically, and financially. Both mothers and fathers experienced burdens in various domains of their lives, with most mothers feeling that the most time was lost in household work, while fathers felt the most time was lost from work due to caring for their sick child; this is consistent with the finding that fewer fathers, compared to mothers, took leave from work at the time of RSV infection (3.3% vs. 25.4%, respectively). Both parents felt emotionally stressed from the child’s condition and prognosis, with sleep deprivation and poorer health quality contributing to the overall burden.

Participants consisted of a mix of those on parental leave and a working population, with more than 35% (68/189) of mothers and 95% (115/120) of fathers actively working at the time of infection (Table 1 footnote). Working caregivers felt an impact on their work, both from missing workdays and from losing motivation to work or feeling anxious about balancing work and caring for their sick child.

The population in this survey shared key characteristics of a modern Japanese family, as the age of caregivers peaks in their 30s, and more than half of all mothers of children younger than or around one year of age are employed either full-time or part-time (including those on parental leave), according to government statistics [20]. Out of the 231 participants who were primary caregivers to their children, 58 (25.1%) were fathers and 173 (74.9%) were mothers, which may reflect how fathers increasingly view themselves as primary caregivers alongside mothers and are involved in child-rearing. The burden specific to caregivers of RSV-infected children who received inpatient care was in line with what has been reported by the Japanese Ministry of Health, Labour and Welfare regarding the burden of family members accompanying a patient for hospital stays, not restricted to a disease type [21]. Similarly, in 2022, a non-profit organization, “Keep Mama Smiling” [22], showed results from a survey indicating that not enough rest or food was secured for the accompanying family member, and not enough explanation was given to the family about what to expect when accompanying the patient [23].

Limitation

This study has several limitations that should be considered when interpreting the findings. First, the RSV severity in children was not assessed, which may influence caregiver burden. Although the sample likely included a range of mild to severe cases, given that RSV is routinely screened in both outpatient and inpatient settings in Japan, this variability could not be quantified and may affect generalizability. Second, the use of a self-administered online panel (Macromill) introduces potential selection bias. Participants may differ systematically from non-participants, particularly in their perception of burden or access to healthcare. And the need for IT literacy may have skewed the sample toward more educated or digitally literate individuals. Although uncommon, responses from multiple caregivers in the same household could also affect response independence. Third, the absence of a control group of caregivers whose children did not contract RSV limits the ability to attribute the reported burden specifically to RSV infection. Other unrelated stressors may have contributed to the responses. Fourth, the interview guide and survey were developed for this study without the use of validated tools or pilot testing, which allowed for tailoring but may limit comparability with other research. Fifth, no statistical testing was conducted, as the study was not designed to compare burden across demographic subgroups. As a result, observed differences cannot be interpreted as statistically significant or free from potential demographic influences. Sixth, reliance on self-reported data may have introduced measurement bias. For instance, some caregivers reported use of RSV prophylaxis despite their child potentially not meeting clinical criteria. This may reflect confusion about prophylaxis options and is further complicated by the lack of data on gestational age and extreme prematurity factors relevant to caregiver burden.

Lastly, recall bias is an inherent limitation of survey-based research. Respondents may have emphasized particularly memorable or emotionally salient experiences, which could skew the overall assessment of the burden.

Key messages

Despite these limitations, the study provides novel insights into the caregiver burden associated with RSV in Japan. It underscores the importance of infection prevention and caregiver support, both of which have implications for child health outcomes and societal productivity. The qualitative approach used to inform questionnaire development enhances the interpretive validity of the findings. Future research should aim to include a non-exposed comparison group and account for key influencing variables better to isolate the impact of RSV infection on caregiver burden.

## Conclusions

Caregivers of children affected by RSV in Japan face a multifaceted burden that encompasses emotional distress, physical exhaustion, and significant financial strain. Among employed caregivers, these challenges are further compounded by disruptions to work productivity and shifts in workplace attitudes. Our findings underscore the importance of recognizing and contextualizing these burdens within the unique cultural and socioeconomic landscape of Japan. A nuanced understanding of caregiver well-being in the context of RSV is essential for informing public health strategies, guiding policy development, and implementing targeted interventions aimed at mitigating the broader impact of RSV on families and the healthcare system.

## Appendices

**Table 7:** Quantitative survey questionnaire (original Japanese version with English summary). ^1^Where 100 represents the sleep duration before child's RSV infection, and 0 represents no sleep at all during the infection period, the same rule applies to health metrics. ^2^Medical expenses refer to costs directly related to treatment, excluding charges for a private room or meals. RSV: respiratory syncytial virus

S.no.	Original Japanese questionnaire	English summary
1	お子さんがRSウイルス感染症に罹患中、あなた自身やご家族の日常生活に普段と異なることはありましたか。	Changes in daily routines due to RSV infection of child
	【身体的】	【Physical】
	睡眠（睡眠の質の低下や睡眠時間の減少など）	Sleep (such as reduced sleep quality or decreased sleep duration)
	一日の生活リズムや家事	Daily routine and household chores
	自身の健康状態（身体の不調、だるさなど）	Personal health (physical discomfort, fatigue, etc.)
	【精神的】	【Emotional】
	家族や友人との関係	Relationship with family and friends
	兄弟姉妹の生活や関係	Relationship with siblings and their daily lives
	職場での人間関係	Relationships at work
	【経済的】	【Economic】
	家庭の経済状況	Family's financial situation
	【仕事や社会進出】	【Work and Career】
	仕事をする時間	Work hours
	仕事の質やパフォーマンス	Work quality and performance
	仕事の継続や働き方	Work continuity and work style
	該当なし	Not applicable
2	お子さんがRSウイルス感染症に罹患中、あなたはどの程度普段と同じように日常生活が送れていましたか。	Extent of change in daily routines
	睡眠の質が低下したり、睡眠時間が減少した	Decreased sleep quality or sleep duration
	とてもそう思う	Totally agree
	どちらかというとそう思う	Somewhat agree
	どちらかというとそう思わない	Somewhat disagree
	全くそう思わない	Totally disagree
	家事など一日の生活リズムが乱れた	Disruption of daily routines, such as house chores
	とてもそう思う	Totally agree
	どちらかというとそう思う	Somewhat agree
	どちらかというとそう思わない	Somewhat disagree
	全くそう思わない	Totally disagree
	身体の不調やだるさなど自身の健康状態が悪化した	Worsened personal health, including physical discomfort and fatigue
	とてもそう思う	Totally agree
	どちらかというとそう思う	Somewhat agree
	どちらかというとそう思わない	Somewhat disagree
	全くそう思わない	Totally disagree
	家族や友人との関係が悪化した	Worsened relationships with family and friends
	とてもそう思う	Totally agree
	どちらかというとそう思う	Somewhat agree
	どちらかというとそう思わない	Somewhat disagree
	全くそう思わない	Totally disagree
	兄弟姉妹の生活リズムが乱れたり、関係が悪化した	Negative impact on siblings' daily routines and relationships
	とてもそう思う	Totally agree
	どちらかというとそう思う	Somewhat agree
	どちらかというとそう思わない	Somewhat disagree
	全くそう思わない	Totally disagree
	職場での人間関係が悪化した	Worsened workplace relationships
	とてもそう思う	Totally agree
	どちらかというとそう思う	Somewhat agree
	どちらかというとそう思わない	Somewhat disagree
	全くそう思わない	Totally disagree
	家庭の経済状況が悪化した	Worsened household financial situation
	とてもそう思う	Totally agree
	どちらかというとそう思う	Somewhat agree
	どちらかというとそう思わない	Somewhat disagree
	全くそう思わない	Totally disagree
	仕事をする時間が削られた	Decreased available work hours
	とてもそう思う	Totally agree
	どちらかというとそう思う	Somewhat agree
	どちらかというとそう思わない	Somewhat disagree
	全くそう思わない	Totally disagree
	仕事の質やパフォーマンスが低下した	Decreased work quality or performance
	とてもそう思う	Totally agree
	どちらかというとそう思う	Somewhat agree
	どちらかというとそう思わない	Somewhat disagree
	全くそう思わない	Totally disagree
	仕事が継続できなかったり、働き方を変えなくてはいけなかった	Inability to continue working or need to change work style
	とてもそう思う	Totally agree
	どちらかというとそう思う	Somewhat agree
	どちらかというとそう思わない	Somewhat disagree
	全くそう思わない	Totally disagree
3.1	お子さんがRSウイルス感染症に罹患中、どのような時間を調整して看病に充てましたか。(1位/番目)	Types of time lost the most from caring for child
	仕事をするための時間	Time for work
	家事をするための時間	Time for household chores
	趣味や自分がゆっくりするための時間	Time for hobbies and personal relaxation
	資格の勉強等、自己啓発のための時間	Time for studying qualifications or self-improvement
	睡眠時間	Sleep time
	兄弟姉妹の世話をする時間	Time for taking care of siblings
	該当なし	Not applicable
3.2	お子さんがRSウイルス感染症に罹患中、どのような時間を調整して看病に充てましたか。(2位/番目)	Types of time lost the second most from caring for child
	仕事をするための時間	Time for work
	家事をするための時間	Time for household chores
	趣味や自分がゆっくりするための時間	Time for hobbies and personal relaxation
	資格の勉強等、自己啓発のための時間	Time for studying qualifications or self-improvement
	睡眠時間	Sleep time
	兄弟姉妹の世話をする時間	Time for taking care of siblings
	該当なし	Not applicable
3.3	お子さんがRSウイルス感染症に罹患中、どのような時間を調整して看病に充てましたか。(3位/番目)	Types of time lost the third most from caring for child
	仕事をするための時間	Time for work
	家事をするための時間	Time for household chores
	趣味や自分がゆっくりするための時間	Time for hobbies and personal relaxation
	資格の勉強等、自己啓発のための時間	Time for studying qualifications or self-improvement
	睡眠時間	Sleep time
	兄弟姉妹の世話をする時間	Time for taking care of siblings
	該当なし	Not applicable
4	お子さんのRSウイルス感染症罹患前の睡眠時間を100（必要な睡眠時間が取れている）とすると、罹患中のあなたの睡眠時間はどの程度でしたか。	^1^Sleep metrics (0-100)
5	お子さんのRSウイルス感染症罹患前のあなたの体調を100（最も良好）として、罹患中のあなたご自身の体調はどの程度でしたか。	Health metrics (0-100)
6	お子さんがRSウイルス感染症に罹患中、あなたの精神的な負担になっていたことは何でしたか。	Causes of distress
	子どもが息苦しそうにしていたこと	Seeing my child struggling to breathe
	咳により子どもが吐き戻したり、体調が安定しなかったこと	My child is experiencing vomiting or instability due to cough
	いつまでこの状態が続くのか分からなかったこと	Not knowing how long this condition would last
	周囲に頼れる人やサポートがなかったこと	Lack of reliable support from people around me
	睡眠不足や疲れからくる精神的な不調	Psychological distress due to sleep deprivation and fatigue
	やる気が出なかったこと	Lack of motivation
	仕事で期待される役割を果たせなかったこと	Inability to fulfill expected roles at work
	（入院の場合）体調の悪い子どもと離れていたこと	(In the case of hospitalization) Being away from my sick child
	該当なし	Not applicable (other)
7	お子さんがRSウイルス感染症に罹患中、あなたが望むサポートを十分に受けられましたか。	Received support
	精神的なサポート（e.g., 何でも相談できる環境、気遣いや声掛けなど）	Psychological support (e.g., having an environment where you can consult about anything, consideration, and encouragement)
	とてもそう思う	Totally agree
	どちらかというとそう思う	Somewhat agree
	どちらかというとそう思わない	Somewhat disagree
	全くそう思わない	Totally disagree
	該当なし	Not applicable
	身体的なサポート（e.g., 料理や洗濯などの家事、子供の世話、あなたの体調面のサポートなど）	Physical support (e.g., household chores such as cooking and laundry, taking care of the child, supporting your physical well-being)
	とてもそう思う	Totally agree
	どちらかというとそう思う	Somewhat agree
	どちらかというとそう思わない	Somewhat disagree
	全くそう思わない	Totally disagree
	該当なし	Not applicable
	経済的なサポート（e.g., 金銭的なサポート、食費、保育費のサポートなど）	Financial support (e.g., in money, food expenses, childcare expenses)
	とてもそう思う	Totally agree
	どちらかというとそう思う	Somewhat agree
	どちらかというとそう思わない	Somewhat disagree
	全くそう思わない	Totally disagree
	該当なし	Not applicable
	仕事のサポート（e.g., 職場や同僚のサポート、周囲から仕事のしやすい環境づくりのサポートなど）	Work support (e.g., from the workplace and colleagues, a work-friendly environment)
	とてもそう思う	Totally agree
	どちらかというとそう思う	Somewhat agree
	どちらかというとそう思わない	Somewhat disagree
	全くそう思わない	Totally disagree
	該当なし	Not applicable
8	お子さんがRSウイルス感染症に罹患中にサポートを受けられた日はどの程度ありましたか。あなたや配偶者のご両親、その他ご家族、ファミリーサポートやシッターなどサポートしてくれた対象者は問いません。	Duration of family support
	1日未満（数時間）	Less than 1 day (a few hours)
	3日以内	Within 3 days
	7日以内	Within 7 days
	それ以上	Over 7 days
	サポートは無かった	No support
9	お子さんがRSウイルス感染症に罹患中に息苦しくしていることで、あなたが強く感じたことを教えてください。	Emotion felt seeing child suffering from RSV infection
	辛そうにしていて可哀そうという気持ち	Feeling sympathy and pity for your child's suffering
	まだ小さいのに病気にさせてしまったと自分を責めてしまう気持ち	Blaming yourself for your child's illness, even though they are still young
	いつまで息苦しさが続くのだろうという不安	Feeling anxious about how long the breathing difficulties will continue
	悪化したらどうしようという不安	Feeling anxious about what will happen if the condition worsens
	他の症状や病気を併発したらどうしようという不安	Feeling anxious about the possibility of other symptoms or complications arising
	誰にも頼れないという孤独感	Feeling a sense of loneliness and feeling like there's no one to rely on
	上記以外	Other
10	お子さんがRSウイルス感染症に罹患したことで、あなたは休職や退職をしたり、仕事と看病を両立するために働き方を変えたり調整したりする必要がありましたか。	Adjustment made to balance work and caring for infected child
	有給や看護休暇を取得した	Took paid leave and nursing leave
	休職した	Took a leave of absence
	退職した	Resigned from the job
	職場を変えた	Changed workplace
	働き方を変えた（短時間勤務や在宅勤務等）	Changed work arrangements (such as working part-time or from home)
	職場や同僚に相談し、融通を利かせてもらった	Consulted with the workplace and colleagues to make adjustments
	何も変わらなかった	No change
11	お子さんがRSウイルス感染症に罹患中、あなたの職場は有給や病欠、看護休暇を含むお休みを取りやすい環境でしたか。	Ease of taking leave in the workplace
	とてもそう思う	Totally agree
	どちらかというとそう思う	Somewhat agree
	どちらかというとそう思わない	Somewhat disagree
	全くそう思わない	Totally disagree
12	お子さんがRSウイルス感染症に罹患中、あなたはお仕事を休まなくてはならない、もしくは仕事を休めないために精神的な負担を感じましたか。	Mentally burdened from taking leave or being unable to take leave
	とてもそう思う	Totally agree
	どちらかというとそう思う	Somewhat agree
	どちらかというとそう思わない	Somewhat disagree
	全くそう思わない	Totally disagree
13	お子さんがRSウイルス感染症に罹患したことで、仕事や働くことに対してあなたの意識の変化はありましたか。	Changes in perception or attitude towards work
	働くことに対しやる気が起こらなくなった	I lost motivation for work
	仕事を続けることがつらく感じた	Continuing with work felt difficult
	責任ある立場に就くことや主要な役割を引き受けることに消極的になった	I became hesitant to take on responsible positions or major roles
	育児と仕事の両立に対して不安になった	I became anxious about balancing parenting and work
	該当なし	Not applicable
14	RSウイルス感染症に罹患したお子さんの治療費に自己負担はありましたか。（直接治療にかかった費用をお考え下さい。個室代や食事代などの間接的な費用は含みません。）	Out-of-pocket direct medical expenses^2^
	あった	Yes
	なかった	No
15	お子さんがRSウイルス感染症に罹患したことで、治療費以外の予期せぬ支出はありましたか。	Unexpected indirect expenses other than direct medical expense
	入院した際の個室代	Hospital private room fee
	入院中の保護者の食費	Caregiver's meal expenses during hospital stay
	ネブライザ―（吸入器）など医療機器の購入	Purchase of medical equipment such as a nebulizer
	病児保育代	Childcare expenses for the sick child
	兄弟姉妹の保育代	Childcare expenses for siblings
	家事のアウトソーシング代	Outsourcing expenses for household chores
	お子さんの治療に伴い発生した、付き添いの保護者及び患児本人以外の食費	Food expenses for individuals other than the accompanying caregivers and sick child
	上記以外の支出	Other
	想定外の支出はなかった	No unexpected expenses
16	RSウイルス感染症に罹患したお子さんの看病でお仕事をお休みされ、あなたご自身または世帯での収入が減少しましたか。	Income decreases due to RSV infection of child
	とてもそう思う	Totally agree
	どちらかというとそう思う	Somewhat agree
	どちらかというとそう思わない	Somewhat disagree
	全くそう思わない	Totally disagree
17	お子さんがRSウイルス感染症に罹患し、入院したことで、あなたご自身が負担に感じたことを教えてください。	Caregiver burden during hospitalization
	付き添い入院中の自身の食事の準備	Meal preparation while accompanying child upon hospital stay
	添い寝や簡易ベッドしか無く、しっかりと眠れなかったこと	Unable to sleep well from sharing a bed or using makeshift bed
	付き添い入院中の罹患児の兄弟姉妹の生活が心配だった	Worrying about siblings while accompanying hospital stay
	家事ができなかったこと	Unable to manage household chores
	子どもが苦しそうだったこと	Seeing the child in distress
	医師や看護師とのコミュニケーションがうまく取れなかったこと	Difficulty in communication with doctors and nurses
	いつまで入院が続くのか不安だったこと	Anxiety about the duration of the hospitalization
	入院中の費用も含めたお金の不安	Financial concerns, including hospitalization costs
	職場や仕事への不安	Anxiety about the workplace and job
	入院している子どもに会えなかったこと	Unable to meet the child who is hospitalized
	該当なし	Not applicable
18	お子さんがRSウイルス感染症に罹患し、外来を受診もしくは退院後の継続通院中に、あなたご自身が負担に感じたことを教えてください。	Caregiver burden during follow-up visits post-discharge
	入院させたいのにできなかった	Unable to hospitalize the child despite wanting to do so
	通院が物理的にもしくは精神的に大変だった	Outpatient visits were physically or mentally challenging
	通院にかかる費用がかさんだ	The cost of outpatient visits became burdensome
	子どもを自宅で看て急変したらどうしようかと不安だった	Anxiety of sudden change while caring for the child at home
	いつまで症状が続くのか不安だった	Uncertain about the duration of the symptoms
	同居する家族にうつることが不安だった	Anxiety from risk of transmitting disease to family members
	通院に付き添うため罹患児の兄弟姉妹の生活への影響が心配だった	Concern about the impact on siblings' lives by accompanying child on outpatient visits
	該当なし	Not applicable
19	お子さんがRSウイルス感染症に罹患したことで、あなたのご家庭で最も負担が大きかったのはどなただと感じますか。	Family member with the greatest burden from RSV infection of child
	父親	Father
	母親	Mother
	祖父母	Grandparents
	同居する兄弟姉妹	Siblings living together
	その他	Other
20	あなたご自身の経験から、RSウイルス感染症の予防薬は必要だと思いますか。	Need for preventive medication
	とてもそう思う	Totally agree
	どちらかというとそう思う	Somewhat agree
	どちらかというとそう思わない	Somewhat disagree
	全くそう思わない	Totally disagree
21	お子さんがRSウイルス感染症に罹患する前に、予防薬が投与できるのであれば受けてみたかったと思いますか。	Willingness to receiving preventive medication
	とてもそう思う	Totally agree
	どちらかというとそう思う	Somewhat agree
	どちらかというとそう思わない	Somewhat disagree
	全くそう思わない	Totally disagree
22	もしRSウイルス感染症の予防薬が投与できるのであれば、あなたが最も抑えたいと思う症状は何ですか。	Preferred symptoms to be relieved by preventive medication
	咳症状	Cough
	息苦しさ	Difficulty in breathing
	肺炎などの重症化	Severe complications such as pneumonia
	熱	Fever
	該当なし	Other
23	もしRSウイルス感染症の予防薬が投与できるのであれば、どの様な予防薬であれば積極的に受けてみたかったと思いますか。	Features of ideal preventive medication
	小児の定期予防接種に含まれているため、自己負担は無し	Covered in routine pediatric vaccinations, no out-of-pocket cost
	とても受けてみたい	Very much want to receive
	受けてみたい	Want to receive
	受けてみたいと思わない	Do not want to receive
	全く受けてみたいと思わない	Do not want to receive at all
	定期予防接種には含まれていないが、国や自治体からの一部接種費用の助成がある	Not covered in routine vaccination, partially subsidized by government or local authorities
	とても受けてみたい	Very much want to receive
	受けてみたい	Want to receive
	受けてみたいと思わない	Do not want to receive
	全く受けてみたいと思わない	Do not want to receive at all
	任意接種で、全額自己負担	Voluntary vaccination, full cost borne by individual
	とても受けてみたい	Very much want to receive
	受けてみたい	Want to receive
	受けてみたいと思わない	Do not want to receive
	全く受けてみたいと思わない	Do not want to receive at all
	かかりつけの病院でポスター等の掲示がある	Recommended by posters in your regular hospital
	とても受けてみたい	Very much want to receive
	受けてみたい	Want to receive
	受けてみたいと思わない	Do not want to receive
	全く受けてみたいと思わない	Do not want to receive at all
	かかりつけ医に勧められた	Recommended by your regular doctor
	とても受けてみたい	Very much want to receive
	受けてみたい	Want to receive
	受けてみたいと思わない	Do not want to receive
	全く受けてみたいと思わない	Do not want to receive at all
	メディア（テレビ・雑誌など）で効果があると取り上げられている	Frequently covered in the media about effectiveness (TV, magazines, etc.)
	とても受けてみたい	Very much want to receive
	受けてみたい	Want to receive
	受けてみたいと思わない	Do not want to receive
	全く受けてみたいと思わない	Do not want to receive at all
	SNSで効果があると取り上げられている	Frequently discussed on social media about effectiveness
	とても受けてみたい	Very much want to receive
	受けてみたい	Want to receive
	受けてみたいと思わない	Do not want to receive
	全く受けてみたいと思わない	Do not want to receive at all
	ママ友との会話で効果があると取り上げられている	Frequently discussed with other moms about effectiveness
	とても受けてみたい	Very much want to receive
	受けてみたい	Want to receive
	受けてみたいと思わない	Do not want to receive
	全く受けてみたいと思わない	Do not want to receive at all
24	お子さんがRSウイルス感染症に罹患したことで、あったら良かったな、利用したかったな、とあなたが思う自治体のサービスは何でしたか。	Need of support services at municipal level
	受診のタイミングを相談できるサービス	Service to consult about the timing of medical visits
	自宅への食事のデリバリー	Meal delivery service to your home
	入院中の病児への付き添い	Accompanying services for sick children during hospitalization
	家事のアウトソーシング	Outsourcing services for household chores
	病児保育	Childcare services for sick children
	兄弟姉妹の託児サービス	Care service for siblings
	該当なし	Not applicable
